# Storage and Release of Spermatozoa from the Pre-Uterine Tube Reservoir

**DOI:** 10.1371/journal.pone.0057006

**Published:** 2013-02-25

**Authors:** Sarah L. Freeman, Gary C.W. England

**Affiliations:** Division of Veterinary Surgery, School of Veterinary Medicine and Science, Faculty of Medicine and Health Sciences, University of Nottingham, Sutton Bonington Campus, Leicestershire, United Kingdom; Institute of Zoology, Chinese Academy of Sciences, China

## Abstract

In mammals, after coitus a small number of spermatozoa enter the uterine tube and following attachment to uterine tube epithelium are arrested in a non-capacitated state until peri-ovulatory signalling induces their detachment. Whilst awaiting release low numbers of spermatozoa continually detach from the epithelium and the uterine tube reservoir risks depletion. There is evidence of attachment of spermatozoa to uterine epithelium in several species which might form a potential pre-uterine tube reservoir. In this study we demonstrate that: (1) dog spermatozoa attach to uterine epithelium and maintain flagellar activity, (2) in non-capacitating conditions spermatozoa progressively detach with a variety of motility characteristics, (3) attachment is not influenced by epithelial changes occurring around ovulation, (4) attachment to uterine epithelium slows capacitation, (5) capacitated spermatozoa have reduced ability to attach to uterine epithelium, (6) under capacitating conditions increased numbers of spermatozoa detach and exhibit transitional and hyperactive motility which differ to those seen in non-capacitating conditions, (7) detachment of spermatozoa and motility changes can be induced by post-ovulation but not pre-ovulation uterine tube flush fluid and by components of follicular fluid and solubilised zona pellucida, (8) prolonged culture does not change the nature of the progressive detachment seen in non-capacitating conditions nor the potential for increased detachment in capacitating conditions. We postulate that in some species binding of spermatozoa to uterine epithelium is an important component of the transport of spermatozoa. Before ovulation low numbers of spermatozoa continually detach, including those which are non-capacitated with fast forward progressive motility allowing the re-population of the uterine tube, whilst around the time of ovulation, signalling from as-yet unknown factors associated with follicular fluid, oocytes and uterine tube secretion promotes the detachment of large numbers of capacitated spermatozoa with hyperactive motility that may contribute to the fertilising pool.

## Introduction

In mammals there is a significant body of information about the attachment of spermatozoa to epithelium of the uterine tube, and how this attachment and the subsequent detachment around the time of ovulation forms an important reservoir for spermatozoa [1]–[3]. Importantly though, whilst it is well established that attachment of spermatozoa to uterine tube epithelium maintains their fertile life by delaying capacitation until ovulation-associated signalling triggers detachment, it is equally clear that low numbers of spermatozoa continually detach prior to any such signal [4]–[6]. Given that only small numbers of spermatozoa enter the uterine tube, it follows that the uterine tube sperm reservoir will ultimately be depleted especially when there is a long interval between mating and ovulation. Interestingly however, pregnancy data from species in which such a long interval is common demonstrate a very limited decline in fertility [7]–[9], indeed in some species motile spermatozoa may be found within the uterus more than one week after mating [10]. In these species and others it has been proposed that the uterus may form an important reservoir of spermatozoa [11]–[15]; presumably allowing for replacement of spermatozoa that are lost from the uterine tube reservoir. Despite the fact that attachment of spermatozoa within uterine glands and crypts has been demonstrated in histological studies [9], [12], [13], [16], there is no information about the nature of such attachment nor whether spermatozoa subsequently detach and if so what mechanisms are involved and motility characteristics are exhibited.

The domestic bitch offers a useful model for investigation of sperm reservoirs since fertile matings may occur up to 7 days before ovulation [10]; resulting in a fertile period of 9 days because fertilisation does not occur until oocytes complete the first meiotic division approximately 48 hours after ovulation [17], [18]. In the bitch, small numbers of spermatozoa attach to uterine tube epithelium and attachment prolongs the fertile life of spermatozoa [19], [20] by delaying capacitation as observed in other species [21]–[23]. Whilst little is known of the mechanisms for induction of spermatozoal detachment from the uterine tube epithelium, capacitation of dog spermatozoa is induced by oestrous oviductal fluid [24], likely mediated by glycosaminoglycans secreted from uterine tube epithelial cells [25] or possibly by factors associated with cumulus cells [26]. It is however not clear how re-population of the uterine tube reservoir might occur during this prolonged period of time.

The aims of this study were to investigate the interaction of dog spermatozoa with uterine epithelium in order to investigate the potential of the uterus to form a pre-uterine tube reservoir. To this aim we conducted a series of in vitro experiments to: (1) study motility characteristics of spermatozoa before attachment and after detachment, (2) establish whether attachment sites changed during oestrus, (3) understand the relationship between capacitation and the attachment and detachment of spermatozoa, (4) investigate how capacitating conditions, uterine tube fluid and uterine tubal junction fluid influenced detachment of spermatozoa, (5) elucidate the possible role of ovarian and oocyte factors on detachment of spermatozoa, (6) monitor the effect of changing the in vitro environment from non-capacitating to capacitating during prolonged culture.

## Materials and Methods

### Ethics

This study was carried out using discarded tissue harvested from clinical cases undergoing routine surgical neutering with owner permission and ethical approval by the School of Veterinary Medicine and Science, University of Nottingham.

### Semen Collection Handling and Evaluation

Semen was collected via glass funnels in the presence of a teaser from 4 healthy male Labrador retrievers of proven fertility. Samples were collected daily as required and subjected to routine evaluation [27]. Throughout the study the percentage progressive motility and the percentage of morphologically normal spermatozoa were greater than 75% and 78% respectively. The sperm-rich components were pooled and sperm concentration was established by haemocytometer. Samples were divided as necessary and washed twice by centrifugation in either M199 Medium (Sigma-Aldrich), or canine capacitation medium (CCM, [28]) as required. Sperm concentration was adjusted to either 20 or 35×10^6^ spermatozoa/ml as detailed later and sperm were immediately used for study. All sperm samples and media were held at 38°C.

For measurement of sperm motility characteristics, samples were examined at x 200 magnification using a negative phase contrast microscope with a temperature controlled stage and local environmental heating to a temperature of 38 C. Video-taped images were examined both subjectively and using an HST 7V1B computer image analysis system (Hobson Sperm Tracking Systems, Sheffield) where 10 individual motility characteristics were measured for each tracked spermatozoa, allowing each to be assigned to one of 3 different sub populations (fast-forward progressive motility [FFPM], transitional motility [TM], hyperactive motility [HM]) or were recorded as non-motile [NM]) according to standard settings and criteria [29], [30].

Sperm capacitation was assessed using the CTC/Hoechst staining procedure [31] and the resulting slides were evaluated for the percentage of F pattern (non-capacitated), B pattern (capacitated) and AR pattern (acrosome-reacted) spermatozoa.

### Harvesting Tissue from Female Reproductive Tracts

Tissue was collected from bitches undergoing routine ovariohysterectomy for the purpose of surgical neutering at various stages of the oestrous cycle. Cycle stage was estimated from clinical history and morphological examination of the ovaries after removal. Cycle stage was classified as proestrus (attractive to male, sero-sangineous vulval discharge and multiple follicles less than 2 mm diameter), oestrus [pre-ovulation] (attractive, sero-sangineous discharge and follicles 2–8 mm diameter), oestrus [post-ovulation] (attractive, sero-sangineous discharge and cavitated corpora lutea 8–10 mm diameter), luteal (oestrus 6–12 weeks previously and solid corpora lutea) or anoestrus (last oestrus 2–5 months previously and no structures visible within the ovary). Ovaries and a section of tissue containing the uterine tube and proximal uterine horn were dissected and placed into M199 at 38°C and transported to the laboratory.

Uterine epithelial explants were collected following longitudinal incision along the length of the proximal uterine horn allowing the endometrial surface to be exposed. Uterine epithelial cell mucosa from the proximal uterine horn was isolated from the musculature and cut into 1 mm^2^ sections. Explants for culture with spermatozoa were placed into M199 supplemented with 0.632 g/L penicillin and 0.050 g/L streptomycin and used to examine sperm attachment/detachment by aligning the epithelial surface vertically (allowing the observation of the mucosal profiles using light microscopy).

Proestrus explants used to harvest epithelial cells were incubated in 0.25% collagenase for 1 hour before isolating epithelial cells by repeated cycles of centrifugation [6] and then introducing into tissue culture plates containing 200 uL M199 supplemented with penicillin and streptomycin as previously described. Following overnight culture at 38°C microscopic examination was used to confirm that clusters of epithelial cells were attached to the base of the plates and culture fluid was gently poured out of the plates to ensure the epithelial cells were not disturbed before replacing with 200 uL CCM.

Follicular fluid (FF) was collected from bitches that were in proestrus and oestrus by aspiration of large antral follicles using a 23-gauge needle and 0.5 mL insulin syringe. Fluid was stored at −20 C and aliquots were later thawed, pooled and diluted 1∶500 with CCM (termed CCM+FF) before immediate use. Progesterone concentration of this solution was 9.8 ng/ml measured by ELISA [32].

Solubilised zona pellucida (SZP) was prepared from primary oocytes recovered by finely chopping ovaries from either the luteal phase or anoestrus. Oocytes with intact zona pellucida were selected, aliquoted and centrifuged (9,600 g for 5 min). The supernatant was removed and the pellet re-suspended in CCM at 1 oocyte/ml and the oocytes were incubated at 60°C for 1 hr. Following incubation, the heat solubilized oocyte suspension (CCM+SZP) was centrifuged to remove any insoluble material and the supernatant used immediately after cooling to 38°C.

Uterine tube (UT) and uterine tubal junction (UTJ) flush fluid was collected as follows. One UT and UTZ/proximal uterine horn (UH) was used for collection of fluid, whilst the contralateral tissue was used for other studies. The UT was transected 5 mm proximal to its insertion into the uterus. UT flushings were collected by inserting a 20-gauge intravenous catheter attached to a 1 mL insulin syringe into the lumen of the ampulla via the infundibulum [24], [25] and fluid was collected at the transected isthmus. UTJ/UH flushing were collected by inserting a new catheter into the transected uterine tube and flushing fluid into the proximal uterine horn. In each case 1.0 mL M199 was slowly injected and fluid was collected into test tubes before aliquoting and storing at −20°C before thawing and pooling fluid from 4 bitches at the same stage of the cycle immediately prior to use.

### Experiment 1: Observations of Sperm Attachment and Detachment from Uterine Epithelium

Observations of sperm attachment and detachment were made on separate days using 4 uterine epithelial explants each from 2 bitches in proestrus and 2 bitches in oestrus [pre-ovulation] that were placed into tissue culture plates containing 200 uL of M199 and allowed to equilibrate at 38°C in 5% CO_2_ in air whilst sperm were prepared. 200 uL of pooled sperm at 20×10^6^/ml suspended in M199 was added to the culture plates and a control plate containing no explants to a final sperm concentration of 10×10^6^/ml. The interaction of sperm with the epithelium in selected areas was video recorded over a period of 10 minutes immediately and every hour for 4 hours to allow later subjective evaluation of attachment of spermatozoa and the motility characteristics of detached sperm. The number of sperm detaching within a given low power microscopic field were recorded during each observation time point. Assessment of the individual motility characteristics of sperm was made immediately after inoculation into the culture plate containing no explants (control) and of all sperm recorded detaching from the epithelium over 30 minutes commencing after 4 hours incubation (detached sperm).

### Experiment 2: Sperm Attachment to Pre-ovulatory and Post-ovulatory Uterine Epithelium

In order to investigate whether attachment to uterine epithelium was influenced by stage of the oestrous cycle around the time of ovulation, 8 uterine epithelial explants each from 2 bitches in oestrus [pre-ovulation], and 2 bitches in oestrus [post-ovulation] were placed into tissue culture plates containing 200 uL M199. Pooled sperm at 35×10^6^/ml suspended in M199 was loaded with fluorescent dye by adding 0.1 mg/ml Hoechst 33342 (Sigma-Aldrich) and after 1 min was diluted in M199 and immediately transferred to the explant cultures to a final concentration of 10×10^6^ spermatozoa/ml and incubated at 38°C in 5% CO_2_ in air for 30 min. Fluid was then removed, explants were washed 6 times in M199 and fixed in 2 mL 3% glutaraldehyde in 0.1 M phosphate buffer (BDH Laboratories) before examination at 400×magnification under ultra-violet illumination (excitation 330–380 nm, emission 420 nm) and the number of blue stained (attached) spermatozoa was counted in three 0.0324 mm^2^ areas of each explant [33]. The number of spermatozoa attached to 1 mm^2^ epithelium was adjusted according to the concentration of live spermatozoa added.

### Experiment 3: Influence of Sperm Attachment on Capacitation Status

To investigate the impact of attachment of spermatozoa to uterine epithelium on capacitation status, spermatozoa were incubated in capacitating conditions in the presence and absence of isolated proestrus epithelial cells. On 4 separate days 200 uL of pooled sperm at 20×10^6^/ml suspended in CCM was added to culture plates containing either: (a) 200 uL CCM, (b) 200 uL CCM with isolated proestrus uterine epithelial cells seeded at the plate base. Final sperm concentrations were 10×10^6^/ml. Each treatment was incubated at 38 C in 5% CO_2_ in air for 6 hours when the plates were subjectively examined microscopically and then vigorously pipetted for 2 minutes before samples were subjected to staining with CTC/Hoechst to establish the capacitation status of sperm.

### Experiment 4: Effect of Capacitation on Sperm Attachment

To investigate the effect of capacitation on sperm attachment, 8 uterine epithelial explants were collect from each of 4 bitches in proestrus and were placed into tissue culture plates with 2 explants per plate within 200 uL of M199. Sperm were prepared by pooling and dividing into two aliquots that were diluted to 35×10^6^/ml with either (a) M199 (non-capacitated) or (b) CCM (capacitated) and incubated at 38°C in 5% CO_2_ in air for 6 hours before being labelled with Hoechst 33342, and adding to explants at 10×10^6^ spermatozoa/ml and further cultured for 30 minutes before fixation and counting the number of spermatozoa attached to 1 mm^2^ to uterine epithelium as previously described.

### Experiment 5: Effect of Capacitating Conditions and Fluid Flushed from the Uterine Tube and Utero-tubal Junction at Various Stages of the Oestrous Cycle on Capacitation Status and Motility Characteristics of Sperm Detaching from Uterine Epithelium

To investigate the effect of fluid flushed from the UT and UTJ on capacitation of spermatozoa and upon motility characteristics of detaching spermatozoa two components to this experiment were conducted.

On 4 separate days 200 uL pooled sperm at 20×10^6^/ml suspended in M199 was added to culture plates containing 200 uL of either : (a) M199 (non-capacitating control), (b) CCM (capacitating), (c) proestrus UT flush fluid, (d) proestrus UTJ/UH flush fluid, (e) oestrus [post ovulation] UT flush fluid, (f) oestrus [post-ovulation] UTJ/UH flush fluid, (g) luteal UT flush fluid, (h) luteal UTJ/UH flush fluid. Each treatment was incubated at 38°C in 5% CO_2_ in air for 6 hours before samples were subjected to staining with CTC/Hoechst to establish the capacitation status of sperm.

In the second part of the experiment 32 uterine epithelial explants were collected from each of 6 bitches in proestrus on separate days and placed in groups of 2 into tissue culture plates containing 200 uL M199. To each plate 200 uL of pooled sperm at 20×10^6^/ml suspended in M199 was added and plates were incubated at 38°C in 5% CO_2_ in air for 4 hours. Explants were removed, rinsed twice in fresh M199 and placed into new tissue wells containing one of eight treatments (2 plates per treatment) each containing 200 ul of either: (a) M199 (non-capacitating control), (b) CCM (capacitating), (c) proestrus UT flush fluid, (d) proestrus UTJ/UH flush fluid, (e) oestrus [post ovulation] UT flush fluid, (f) oestrus [post-ovulation] UTJ/UH flush fluid, (g) luteal UT flush fluid, (h) luteal UTJ/UH flush fluid. After 1 hour further incubation, selected areas of epithelium where spermatozoa were noted to be detaching were video recorded (observations occurred over a period of a further 160 minutes with 2×10 minutes of recording for each treatment plate undertaken in random predetermined order over the 6 days of the experiment), and each plate was then visually searched and the number of detached non-motile (NNM) sperm was counted. Video recordings were later subjected to computer image analysis to enable identification of individual sperm motility characteristics of detached spermatozoa enabling the percentage of FFPM, TM and HM to be measured and the number of spermatozoa detaching within 10 minutes to be counted.

### Experiment 6: Effect of Follicular Fluid and Solubilised Zona Pellucida on Capacitation Status and Motility Characteristics of Sperm Detaching from UtErine Epithelium

In attempt to further elucidate the effects of oestrus [post-ovulation] flush fluid on detachment of spermatozoa from uterine epithelium two components to this experiment were conducted.

On 4 separate days 200 uL pooled sperm at 20×10^6^/ml suspended in M199 was added to culture plates containing 200 uL of either: (a) M199 (non-capacitating control), (b) CCM (capacitating), (c) oestrus [post ovulation] UT flush fluid, (d) CCM+FF (as previously described), (e) CCM+SZP (as previously described). Each treatment was incubated at 38 C in 5% CO_2_ in air for 6 hours before samples were subjected to staining with CTC/Hoechst to establish the capacitation status of sperm.

In the second part of the experiment, 10 uterine epithelial explants were collected from each of 6 bitches in proestrus on separate days and placed in groups of 2 into tissue culture plates containing 200 uL M199. To each plate 200 uL of pooled sperm at 20×10^6^/ml suspended in M199 was added and plates were incubated at 38°C in 5% CO_2_ in air for 4 hours. Explants were removed, rinsed twice in fresh M199 and placed into new tissue wells containing one of five treatments (2 plates per treatment) each containing 200 ul of either: (a) M199 (non-capacitating control), (b) CCM (capacitating), (c) oestrus [post ovulation] UT flush fluid, (d) CCM+FF (as previously described), (e) CCM+SZP (as previously described).

After 1 hour further incubation, selected areas of epithelium where sperm were noted to be detaching were video recorded over 60 minutes (2×10 minutes of recording for each treatment plate undertaken in random predetermined order over the 6 days of the experiment), after which each plate was visually searched and the number of detached and non-motile (NNM) sperm was counted. Video recordings were later subjected to computer image analysis to enable identification of individual sperm motility characteristics of detached spermatozoa enabling the percentage of FFPM, TM and HM to be calculated, and to allow the number of spermatozoa detaching within 10 minutes to be counted.

### Experiment 7: Sperm Motility Characteristics at Detachment in Capacitating and Non-Capacitating Conditions during Prolonged Incubation

In order to investigate the effect of prolonged culture on detachment of spermatozoa, 16 uterine epithelial explants were collected from each of 6 bitches in proestrus on separate days and placed in groups of 2 into tissue culture plates containing 200 uL M199. To each plate 200 uL of pooled sperm at 20×10^6^/ml suspended in M199 was added and plates were incubated at 38°C in 5% CO2 in air for 4 different times (4 plates per time period) either: (a) 4 hours, (b) 24 hours, (c) 48 hours, (d) 96 hours. At the end of the appropriate incubation period explants were removed rinsed twice in fresh M199 and placed into new tissue wells containing one of two treatments (2 plates per treatment): (a) 200 uL M199 (non-capacitating control), (b) 200 uL CCM+SZP (as previously described). CCM+SZP was used as the inducer of sperm detachment in this study because of limited availability of FF. After 1 hour of further incubation, selected areas of epithelium where sperm were noted to be detaching were video recorded over 60 minutes (2×10 minutes of recording for each treatment plate undertaken in random predetermined order over the 6 days of the experiment). The percentage of detached spermatozoa that exhibited FFPM, TM and HM were measured, the number of spermatozoa detaching within 10 minutes was counted, and plate were search for non-motile sperm (NNM) as previously described.

### Statistical Analysis

Means (± S.E.M.) and percentages were calculated for each group, and data were analysed using either the t-test or one-way analysis of variance with Tukey’s post hoc test (InStat3, GraphPad). Values were considered to be significant with P<0.05.

## Results

### Experiment 1: Observations of Sperm Attachment and Detachment from Uterine Epithelium

**Figure 1:**
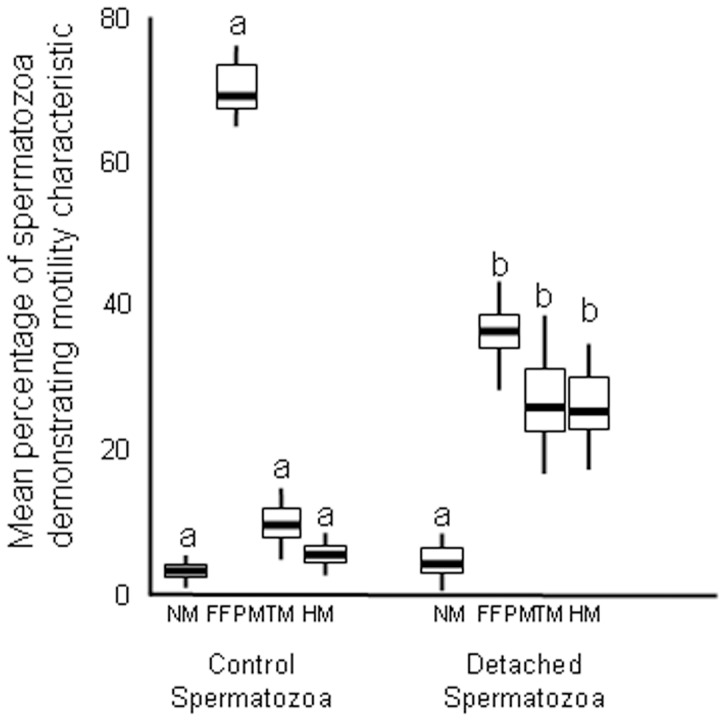
Motility characteristics of spermatozoa before attachment and after detachment from uterine epithelium. Sperm samples diluted in M199 were incubated with uterine epithelial explants from bitches in proestrus and oestrus [pre-ovulation]. Data are mean percentage (± S.E.M.) motility characteristics (NM; non-motile, FFPM; fast forward progressive motility, TM; transitional motility, HM; hyperactive motility) at 0 hours for controls (not incubated with explants) and over 4.0–4.5 hours for detaching spermatozoa. Comparisons are individual motility characteristics between treatments; means with a letter in common are not significantly different p<0.05.

Control spermatozoa had a high percentage of FFPM motility and low percentage of TM and HM (Figure 1). Within minutes of placing in culture with explants many spermatozoa were attached to uterine epithelial cells by their head whilst their tails maintained active motility. Observation of individual spermatozoa during the 10 minute observation time points each hour showed that the majority of spermatozoa remained attached although a small number had a transient detachment phase before re-attaching to adjacent epithelium. Within 60 minutes the majority of spermatozoa were attached and only small numbers were present in the medium. There was no apparent difference noted in tissue from bitches in either proestrus or oestrus and because the number of samples was small, data from these tissues were combined. The mean numbers of sperm detaching per low power field during the 10 minute time period at 1, 2,3 and 4 hours were not significantly different and were 19.7±1.9 (S.E.M.), 21.8±1.1, 23.4±1.3, and 24.7±1.8 respectively.

At 4 hours incubation the motility characteristics of detaching spermatozoa was significantly reduced for FFPM, and increased for TM and HM compared with control spermatozoa (Figure 1).

### Experiment 2: Sperm Attachment to Pre-ovulatory and Post-ovulatory Uterine Epithelium

The mean number of spermatozoa attached per mm^2^ to oestrus [pre-ovulation] uterine epithelium (4460±240) was not different to the numbers attached mm^2^ to oestrus [post-ovulation] uterine epithelium (4370±230).

### Experiment 3: Influence of Sperm Attachment on Capacitation Status

**Figure 2:**
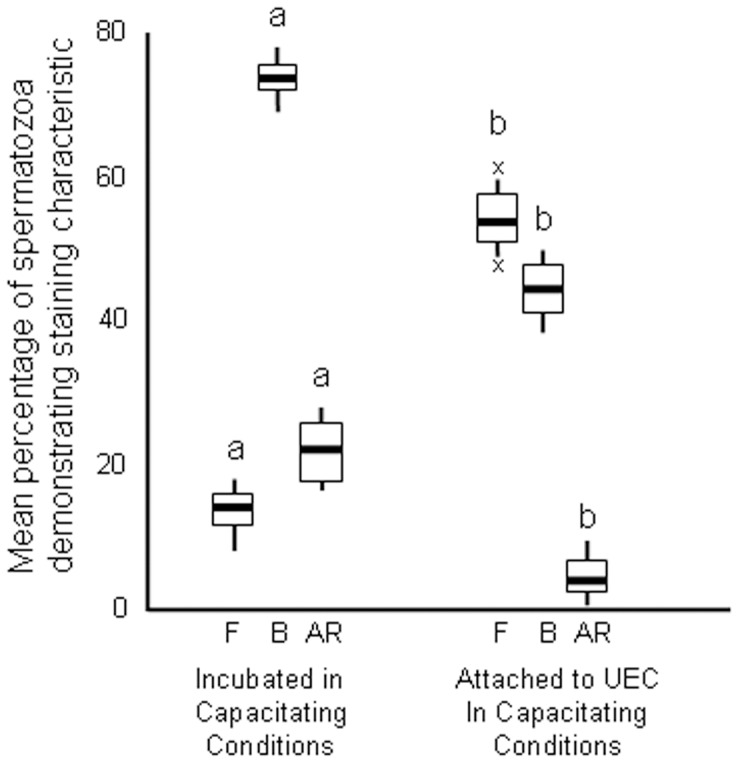
Chlortetracycline staining characteristics in capacitating conditions of spermatozoa attached to uterine epithelium and non-attached spermatozoa. Sperm samples diluted in canine capacitation medium were incubated without epithelial cells or with isolated proestrus uterine epithelial cells (UEC). Data are mean percentage (± S.E.M.) staining characteristics (F; non-capacitated, B; capacitated, AR; acrosome-reacted) after 6 hours incubation. Comparisons are individual staining characteristics between treatments; means with a letter in common are not significantly different p<0.05.

After 6 hours culture, microscopic examination of the culture plates containing spermatozoa incubated with CCM subjectively demonstrated a predominance of active motility characteristics (TM and HM). When spermatozoa were incubated in CCM with epithelial cells very few spermatozoa were free within the medium (and subjectively these had TM and HM motility characteristics) but the majority of spermatozoa were attached by their heads to uterine epithelial cells but had continued flagellar activity. After pippetting the majority of spermatozoa and epithelial cells were dispersed facilitating assessment of the CTC staining pattern of individual spermatozoa. Where spermatozoa and epithelial cells were clumped and it was not possible to assess CTC staining characteristics these were ignored. For samples incubated in CCM in the absence of epithelial cells there were low numbers of sperm with F-pattern staining (15.4±9.1%) and high numbers of spermatozoa that were capacitated (B-pattern mean 72.4±8.2%) and acrosome reacted (AR-pattern mean 21.3±10.2%). All staining characteristics were significantly different for sperm that were incubated with/attached to uterine epithelial cells (Figure 2); more spermatozoa were F-pattern (54.6±7.4%) whilst fewer sperm were B-pattern (42.1±8.9%) and AR-pattern (6.1±5.2%).

### Experiment 4: Effect of Capacitation on Sperm Attachment

The mean number of non-capacitated spermatozoa attached per mm^2^ to uterine epithelium (4386±211) was significantly greater than the number of capacitated spermatozoa attached mm^2^ to uterine epithelium (2670±486).

### Experiment 5: Effect of Capacitating Conditions and Fluid Flushed from the Uterine Tube and Uterus at Various Stages of the Oestrous Cycle on Capacitation Status and Motility Characteristics of Sperm Detaching from Uterine Epithelium

**Figure 3:**
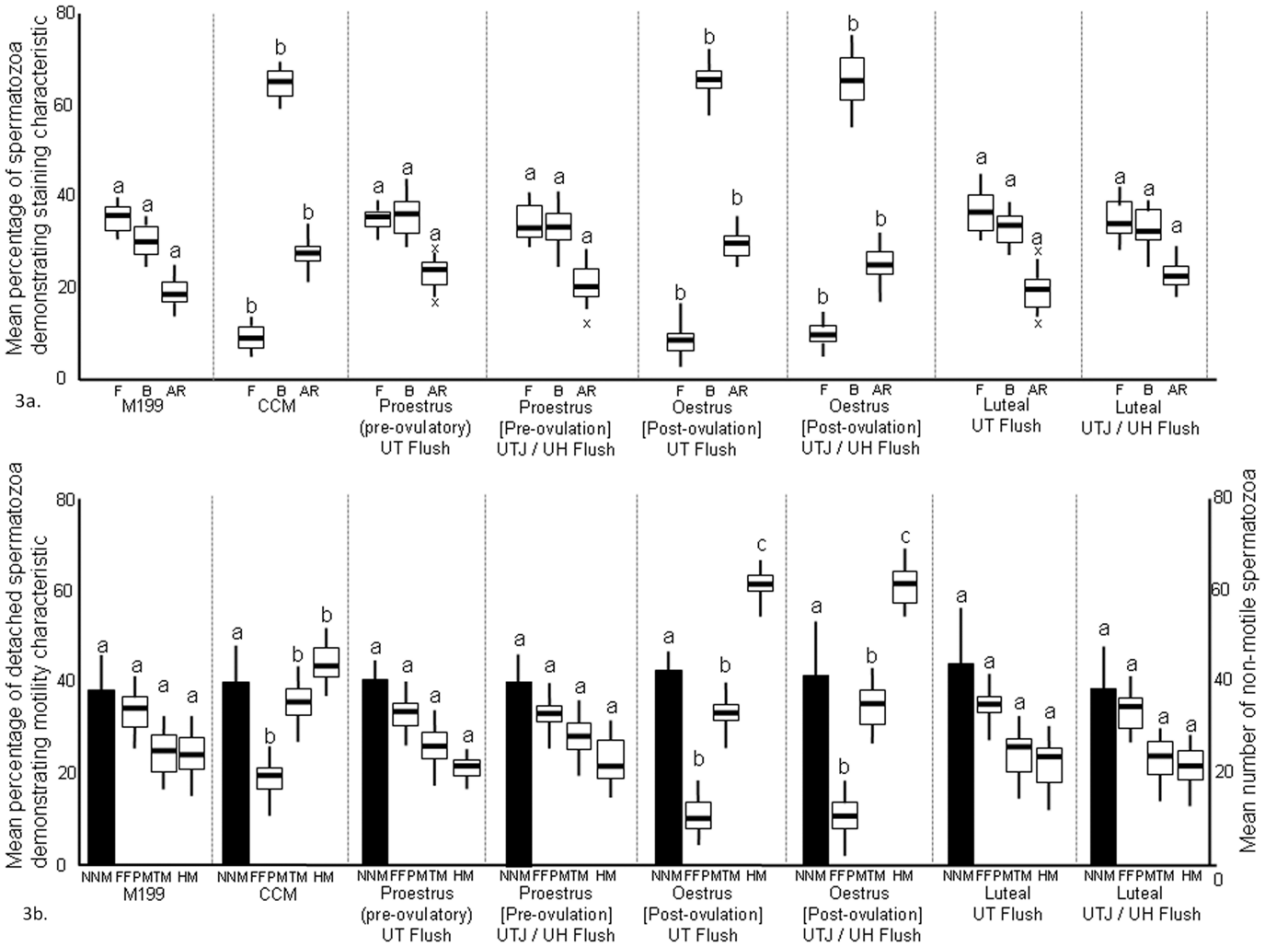
a. Chlortetracycline staining characteristics of spermatozoa after exposure to reproductive tract flush fluid. Sperm samples diluted with M199 were diluted with (a) non-capacitating control (M199), (b) capacitation medium (CCM), (c) proestrus (pre-ovulatory) uterine tube flush fluid (UT Flush), (d) proestrus (pre-ovulatory) uterine tubal junction/uterine horn flush fluid (UTJ/UH Flush), (e) oestrus [post-ovulation] uterine tube flush fluid, (f) oestrus [post-ovulation] uterine tubal junction/uterine horn flush fluid, (g) luteal uterine tube flush fluid, (h) luteal uterine tubal junction/uterine horn flush fluid. Data are mean percentage (± S.E.M.) staining characteristics (F; non-capacitated, B; capacitated, AR; acrosome-reacted) after 6 hours incubation. Comparisons are individual staining characteristics between treatments; means with a letter in common are not significantly different p<0.05. b. Motility characteristics of spermatozoa detaching from uterine epithelium after exposure to reproductive tract flush fluid. Sperm samples diluted with M199 were incubated with proestrus uterine epithelial explants for 4 hours before transfer to new medium which was (a) non-capacitating control (M199), (b) capacitation medium (CCM), (c) proestrus (pre-ovulatory) uterine tube flush fluid (UT Flush), (d) proestrus (pre-ovulatory) uterine tubal junction/uterine horn flush fluid (UTJ/UH Flush), (e) oestrus [post-ovulation] uterine tube flush fluid, (f) oestrus [post-ovulation] uterine tubal junction/uterine horn flush fluid, (g) luteal uterine tube flush fluid, (h) luteal uterine tubal junction/uterine horn flush fluid. Data are mean number (± S.E.M.) of non-motile spermatozoa per plate (NNM) and mean percentage (± S.E.M.) motility characteristics (FFPM; fast forward progressive motility, TM; transitional motility, HM; hyperactive motility) of detaching spermatozoa over 1.0–3.5 hours culture after change of the media. Comparisons are individual motility characteristics between treatments; means with a letter in common are not significantly different p<0.05.

In the first part of this experiment, CCM produced a significant increase in B-pattern and AR-pattern spermatozoa and a decrease in F-pattern spermatozoa compared with the M199 controls (Figure 3a). Proestrus (pre-ovulatory) flush fluid from either UT or UTJ/UH and luteal flush fluid from either UT or UTJ/UH produced no significant change in capacitation status compared with the M199 control, whilst oestrus [post-ovulation] flush fluid from either UT or UTJ/UH produced a significant effect that was not different to CCM (Figure 3a).

In the second part of the experiment, spermatozoa were observed detaching from uterine epithelial explants after 5 hours incubation in M199 (non-capacitating) conditions. Sperm of all motility characteristics were detected; FFP (37.2±9.1%), TM (25.4±11.2%), HM (24.1±6.4%). These results were similar to those of Experiment 1 when sperm-explant cultures were undertaken in non-capacitating conditions.

Transfer of explants to CCM (capacitating conditions) resulted in a significant increase in the number of spermatozoa detaching within 10 minutes (43.4±2.1) compared with those detaching in the M199 non-capacitating control (21.7±1.8). The motility characteristics of the detaching spermatozoa was also significantly different; there were fewer FFPM spermatozoa and increased percentages of TM and HM spermatozoa (Figure 3b).

Proestrus (pre-ovulatory) flush fluid from both the UT and UTJ/UH produced no change in the number of spermatozoa detaching within 10 minutes (19.8±1.8 and 20.4±2.3 for UT and UTJ/UH respectively) compared the the M199 control. Detached spermatozoa had no significant difference in motility characteristics compared with the non-capacitated M199 control (Figure 3b).

Compared with M199, the oestrus [post-ovulation] flush fluid from both the UT and UTJ/UH produced a significant increase in the number of spermatozoa released within 10 minutes (48.2±3.1 and 46.8±3.2 for UT and UTJ/UH respectively); values were not different to CCM. Detached spermatozoa had motility characteristics that were significantly different to the non-capacitated M199 controls; there were significantly fewer FFPM and increased percentages of TM and HM spermatozoa. Significantly greater proportions of HM were found following exposure to oestrus [post-ovulation] flush fluid than CCM.

Luteal flush fluid from both the UT and UTJ/UH produced no change in the number of spermatozoa detached (18.9±2.4), nor the motility characteristics of detached spermatozoa compared with non-capacitated M199 control (Figure 3b).

There was no significant difference in the mean number of non-motile spermatozoa found within the culture plates for any of the treatments (Figure 3b).

### Experiment 6: Effect of Follicular Fluid and Solubilised Zona Pellucida on Capacitation Status and Motility Characteristics of Sperm Detaching from Uterine Epithelium

**Figure 4:**
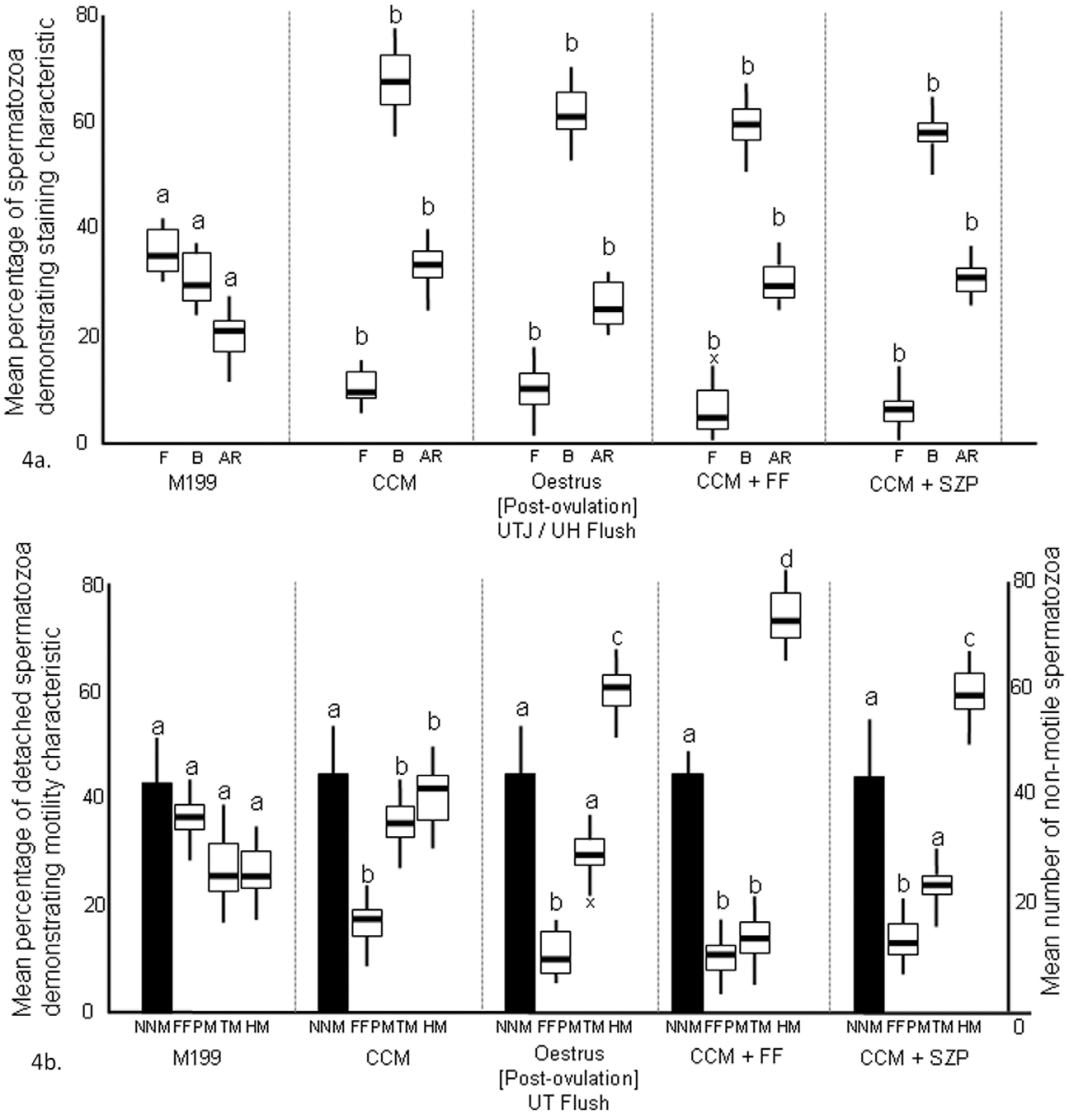
a. Chlortetracycline staining characteristics of spermatozoa after exposure to uterine tube fluid, FF and SZP. Sperm samples diluted with M199 were diluted with (a) non-capacitating control (M199), (b) capacitation medium (CCM), (c) oestrus [post-ovulation] uterine tube flush fluid (UT Flush), (d) follicular fluid in CCM (CCM+FF), (e) solublised zona pellucida in CCM (CCM+SZP). Data are mean percentage (± S.E.M.) staining characteristics (F; non-capacitated, B; capacitated, AR; acrosome-reacted) after 6 hours incubation. Comparisons are individual staining characteristics between treatments; means with a letter in common are not significantly different p<0.05.b. Motility characteristics of spermatozoa detaching from uterine epithelium after exposure to uterine tube fluid, FF and SZP. Sperm samples diluted with M199 were incubated with proestrus uterine epithelial explants for 4 hours before transfer to new medium which was (a) non-capacitating control (M199), (b) capacitation medium (CCM), (c) oestrus [post-ovulation] uterine tube flush fluid (UT Flush), (d) follicular fluid in CCM (CCM+FF), (e) solublised zona pellucida in CCM (CCM+SZP). Data are mean number (± S.E.M.) of non-motile spermatozoa per plate (NNM) and mean percentage (± S.E.M.) motility characteristics (FFPM; fast forward progressive motility, TM; transitional motility, HM; hyperactive motility) of detaching spermatozoa over 1.0–2.5 hours culture after change of the media. Comparisons are individual motility characteristics between treatments; means with a letter in common are not significantly different p<0.05.

In the first part of this experiment, CCM produced a significant increase in B-pattern and AR-pattern spermatozoa and a decrease in F-pattern spermatozoa compared with the M199 controls (Figure 4a). Both oestrus [post-ovulation] flush fluid, CCM+FF and CCM+SZP produced changes in capacitation status that differed from the M199 control but did not differ to that produced by exposure to CCM alone (Figure 4a).

In the second part of the experiment, low numbers of spermatozoa detached from uterine epithelium following incubation in M199 (non-capacitating) (22.6±1.9 spermatozoa per 10 minutes). The detached spermatozoa had a range of motility characteristics; FFP (38.7±10.1%), TM (29.1±12.1%), HM (21.4±7.2%). These results were similar to those of Experiment 5.

Transfer of explants to CCM (capacitating conditions) resulted in a significant increase in the number of spermatozoa released within 10 minutes (43.8±3.8) compared with those released in non-capacitating conditions. Detaching spermatozoa had significantly lower percentages of FFPM spermatozoa, and increased percentages of TM and HM spermatozoa compared with those observed for spermatozoa incubated in M199 (Figure 4b).

Exposure to oestrus [post-ovulation] flush fluid resulted in a significant increase in the number of detaching spermatozoa per 10 minutes (45.2±2.5) compared with M199 controls; numbers detaching were not different to with CCM. The detached spermatozoa had similar motility characteristics to those induced by CCM although there were significantly higher percentages of HM (Figure 4b).

Both FF and SZP in combination with CCM produced an in increased detachment of spermatozoa (48.1±2.3 and 42.9±1.5 spermatozoa detached over 10 minutes for CCM+FF and CCM+SZP respectively). CCM+FF was associated with increased proportions of spermatozoa detaching with HM than either controls, CCM or oestrus [post-ovulatory] flush fluid (Figure 4b). CCM+SZP produced a similar effect to CCM+FF although there were lower percentages of HM spermatozoa which was not significantly different to oestrus [post-ovulation] flush fluid (Figure 4b).

There was no significant difference in the mean number of non-motile spermatozoa found within the culture plates for any of the treatments (Figure 4b).

### Experiment 7: Sperm Motility Characteristics at Detachment in Capacitating and Non-Capacitating Conditions during Prolonged Incubation

**Figure 5:**
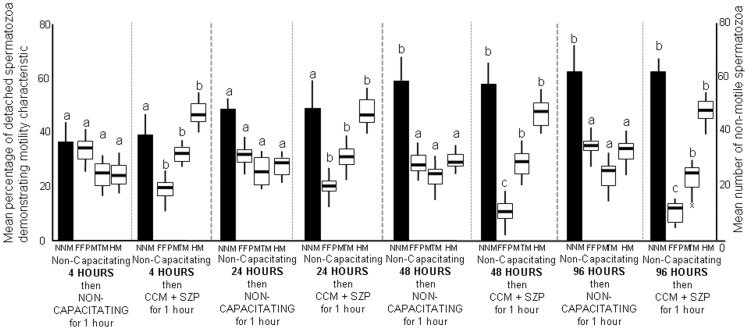
Motility characteristics of spermatozoa detaching from uterine epithelium in non-capacitating and capacitating conditions during prolonged culture. Sperm samples diluted with M199 were incubated with proestrus uterine epithelial explants for either (a) 4 hours, (b) 24 hours, (c) 48 hours, (d) 96 hours before transfer to new medium which was (a) non-capacitating control (M199), (b) solublised zona pellucida in CCM (CCM+SZP). Data are mean number (± S.E.M.) of non-motile spermatozoa per plate (NNM) and mean percentage (± S.E.M.) motility characteristics (FFPM; fast forward progressive motility, TM; transitional motility, HM; hyperactive motility) of detaching spermatozoa over 1.0–2.0 hours culture after change of the media. Comparisons are individual motility characteristics between treatments; means with a letter in common are not significantly different p<0.05.

Following culture in non-capacitating conditions and transfer to non-capacitating conditions at any time point there was no significant change in the mean number of detaching spermatozoa/10 minutes which was 22.0±1.9, 21.2±1.7, 20.4±2.2, 17.2±3.1 for culture at 4, 24, 48 and 96 hours respectively. At all of these times for spermatozoa cultured continually in non-capacitating conditions there was no difference in the motility characteristics of the detaching spermatozoa which had a range of FFPM, TM and HM (Figure 5) similar to Experiments 1, 5 and 6. There was no difference in the mean NNM spermatozoa at 4 and 24 hours culture, but values were significantly higher at 48 and 96 hours culture (Figure 5).

For sperm incubated in M199 and then transferred to CCM+SZP there was a significant increase in the number of sperm detaching/10 minutes at each time point compared with the control at the same time point (49.1±3.1, 47.1±2.4, 44.9±4.2, 44.1±2.8 for 4, 24, 48 and 96 hours culture respectively) but values between these time points were not significantly different to each other. At each time point the detached spermatozoa had significantly greater proportions of TM and HM and lower FFPM than the non-capacitated controls (Figure 5). When exposed to CCM+SZP there were no difference in the motility characteristics of the detached spermatozoa between 4 and 24 hours (Figure 5), and no differences in TM and HM at any of the time points, although lower percentages of FFPM in detached spermatozoa were present after 48 and 96 hours culture. Increased mean numbers of NNM spermatozoa were found after 48 and 96 hours culture (Figure 5).

## Discussion

The transport of spermatozoa within the female reproductive tract differs depending on whether spermatozoa are deposited before or after ovulation [13], [34]. With post-ovulatory mating a large number of spermatozoa progress within the female tract, whilst for pre-ovulatory mating, a small number of spermatozoa enter the uterine tube and attach to uterine tube epithelium [1]–[3], [34]. Low numbers of spermatozoa appear to continually detach from uterine tube epithelium [4]–[6], but overall the attachment of spermatozoa slows the process of capacitation so that the fertile life of spermatozoa is maintained until close to ovulation when they become activated and increasing numbers detach with hyperactive motility [5], [34], [35]. It is not clear how the numbers of spermatozoa within the uterine tube reservoir are maintained particularly when the interval from mating to ovulation is prolonged, although it has been suggested that the uterus provides a store of spermatozoa for the uterine tube [11], [36], [37]; details of how this might be achieved have not previously been described.

Here using non capacitating conditions (that may be unlike those found in vivo [34]), we observed that spermatozoa rapidly attached by their heads to uterine epithelium and had flagella activity. Over 4 hours of culture, a continual low number of spermatozoa detached at an almost constant rate, and when motility of detaching spermatozoa was measured at 4 hours culture approximately 40% of the detaching spermatozoa had FFPM compared with approximately 70% prior to attachment. It is clear that attachment of spermatozoa to uterine epithelium plays a role in modulating sperm motility characteristics. Interestingly, the motility characteristics of detaching spermatozoa were constant throughout periods of prolonged culture in non-capacitating conditions, with approximately 40% of detaching spermatozoa exhibiting FFPM and 60% exhibiting TM and HM. These are the first observations of the behaviour of spermatozoa during periods of prolonged culture with uterine epithelium, and these findings are consistent with our contention that, at least in the bitch where a long interval between semen deposition and fertilisation is common, prolonged periods of storage of spermatozoa may occur by attachment to uterine epithelium [15]. Furthermore, we propose that in non-capacitating conditions there is a progressive release of spermatozoa, where a low number with a variety of motility characteristics detach and this allows continued availability of spermatozoa to replace the uterine tube reservoir. These contentions are supported by previous observations of the attachment and distribution of spermatozoa within the bitch’s reproductive tract [15], [16], and earlier work where disappearance of spermatozoa from uterine glands prior to the loss of spermatozoa from the uterine lumen suggested that over time glandular spermatozoa supplement the luminal population [10].

These experiments also demonstrated that in non-capacitating conditions there was no significant change in the ability of spermatozoa to attach to uterine epithelium in the peri-ovulatory period. Presumably any changes in attachment of spermatozoa that may occur in the peri-ovulatory period are not influenced by the availability of binding sites for spermatozoa, similar to observations relating to the uterine tube in other species [38]. Furthermore, we found that attachment to/association of spermatozoa with proestrus uterine epithelial cells reduced capacitation and acrosome reaction of spermatozoa. In addition, we found that capacitated spermatozoa had reduced ability to attach compared with non-capacitated spermatozoa, similar to the situation with attachment of spermatozoa to uterine tube epithelium [4], [39], [40].

An important feature of this series of experiments was the observation of an approximate 2-fold increase in the number of detaching spermatozoa when culture conditions were changed from non-capacitating to capacitating (using a simple canine capacitation medium [28]). As well as increasing the number of detaching spermatozoa, capacitating conditions produced a significant change in their motility characteristics, with larger proportions of the detaching spermatozoa demonstrating TM and HM than observed for spermatozoa detaching in non-capacitating conditions. Interestingly, exposure to pre-ovulatory UT and UTJ/UH flush fluid from proestrous bitches produced no increase in the numbers of detaching spermatozoa or detectable change in the motility characteristics of detached spermatozoa compared with the controls. The situation with post-ovulatory UT and UTJ/UH flush fluid from oestrous bitches was however considerably different and produced an increase in the number of detaching spermatozoa similar to that produced by CCM. Post-ovulatory flush fluid was associated with a high percentage of detached spermatozoa that had transitional and hyperactive motility. We propose that when conditions in the female reproductive tract change to capacitating, and when an appropriate post-ovulatory signal is present, increased numbers of spermatozoa detach presumably to be available for the fertilising pool. Interestingly, exposure to oestrus [post-ovulatory] flush fluid increased capacitation and acrosome reaction of spermatozoa and it is plausible that this may be associated with changes in motility characteristics that result in detachment of spermatozoa. Previous work has also shown that oestrus uterine tube flush fluid can increase capacitation of dog spermatozoa [24] and that elevated concentrations of glycosaminoglycans present in oestrus uterine tube flush fluid can induce capacitation and hyperactive motility [25]. Our findings, that the uterine sperm reservoir is affected by peri-ovulatory signals in a similar way to the uterine tube reservoir add to the current literature. Further investigations are needed into the mechanisms associated with detachment of spermatozoa, since although changes in motility characteristics of spermatozoa are likely to be important to facilitate detachment, at least for the uterine tube, a variety of interesting biological mechanism have now been detected [36], [41], [42]. In the present study we were able to demonstrate that both follicular fluid and solubilised zona pellucida in the presence of capacitating conditions induced capacitation but not greater than CCM alone, but resulted in a greater proportion of detached spermatozoa exhibiting hyperactive motility than observed in capacitating conditions alone, adding weight to the evidence proposing that detachment of spermatozoa is regulated by peri-ovulatory events. Clearly significant further work is needed into the signals identified by these studies (including examination of follicular fluid and solubilised zona pellucida in non-capacitating conditions) and whether they are observed in vivo, but the results are consistent with observations in other species that capacitation, motility changes and detachment of spermatozoa from uterine tube epithelium can be influenced by follicular, oocyte and hormonal factors [26], [34], [43].

As well as demonstrating that detachment of large numbers of spermatozoa from uterine epithelium may be induced by exposure to capacitating conditions and factors associated with follicular fluid, solubilised zona pellucida and uterine tube flush fluid, in the final experiment we demonstrated that spermatozoa may remain attached to uterine epithelium for a prolonged period of time. Throughout this time a consistent low number of spermatozoa continually detached with a fixed distribution of motility characteristics. We propose that in non-capacitating conditions in the absence of specific peri-ovulatory signalling the uterus provides a reservoir for the slow release of spermatozoa that are available for movement within the reproductive tract, presumably to replace the depleting uterine tube reservoir. When culture conditions were changed to capacitating combined with an ovulation-related signal (here SZP), there was an increase in the number of detaching spermatozoa with a large proportion exhibiting TM and HM. Presumably then the uterine reservoir is able to respond to similar signals as the uterine tube reservoir and result in increased release of spermatozoa given appropriate ovulation-related signalling that allows more spermatozoa to be available for fertilisation.
